# Supplementary material for: A weighted region-based level set method for image segmentation with intensity inhomogeneity
Source: PLoS One. 2021 Aug 19;16(8):e0255948. doi: 10.1371/journal.pone.0255948 (PMC8376002; doi:10.1371/journal.pone.0255948)
Supplement: S1 File — (PDF) [file pone.0255948.s001.pdf]

The description of the data set

1. Figure 1 is a synthetic image
2. The first image of Figure 2 comes from the medical image provided by the cooperative project hospital; the second image of Figure 2 comes from DRIVE data set which is used for retinal image segmentation （https://drive.grand-challenge.org/ ）; the third image of Figure 2 comes from MRI Brain image segmentation dataset （https://www.oasis-brains.org/ ）.
3. The brain images of figure 3 comes from MRI Brain image segmentation dataset(https://www.oasis-brains.org/)
4. The heart images of Figure 4 comes from http://www.cardiacatlas.org/studies/amrg-cardiac-atlas/
5. The images of Figure 5 and 6 come from the Berkeley dataset, http://www.eecs.berkeley.edu/Research/Projects/CS/vision/grouping/segbench/.
